# Nanoparticle containing recombinant excretory/secretory-24 protein of *Haemonchus contortus* enhanced the cellular immune responses in mice

**DOI:** 10.3389/fvets.2024.1470084

**Published:** 2024-11-12

**Authors:** Muhammad Waqqas Hasan, Muhammad Haseeb, Javaid Ali Gadahi, Muhammad Ehsan, Qiangqiang Wang, Shakeel Ahmed Lakho, Ali Haider, Muhammad Tahir Aleem, Kalibixiati Aimulajiang, Mingmin Lu, Lixin Xu, Xiaokai Song, Xiangrui Li, Ruofeng Yan

**Affiliations:** ^1^MOE Joint International Research Laboratory of Animal Health and Food Safety, College of Veterinary Medicine, Nanjing Agricultural University, Nanjing, Jiangsu, China; ^2^Key Laboratory of Molecular Target and Clinical Pharmacology and the State and NMPA Key Laboratory of Respiratory Disease, School of Pharmaceutical Science and The Fifth Affiliated Hospital, Guangzhou Medical University, Guangzhou, China; ^3^State Key Laboratory of Pathogenesis, Prevention and Treatment of High Incidence Diseases in Central Asia, Clinical Medicine Institute, The First Affiliated Hospital of Xinjiang Medical University, Ürümqi, Xinjiang, China

**Keywords:** *Haemonchus contortus*, HcES-24, PLGA and CS nanoparticle, Th1 immune response, mice

## Abstract

*Haemonchus contortus* poses a global challenge as a parasite affecting small ruminants, yet the problem of absence of an effective vaccine against *H. contortus* infection still exists. This investigation sought to appraise the immunological reaction induced by recombinant *H. contortus* excretory/secretory-24 (rHcES-24) in combination with complete Freund’s adjuvant (CFA) and bio-polymeric nanoparticles (NPs) within a murine model. In this study, rHcES-24 was encapsulated in poly(d, l-lactide-co-glycolide) (PLGA) and chitosan (CS) NPs, administered subcutaneously to mice. Researchers analyzed the NPs using scanning electron microscope (SEM) and assessed lymphocyte proliferation, specific antibodies, cytokines, T cell proliferation (CD3e^+^CD4^+^, CD3e^+^CD8a^+^), and phenotypic alteration in splenocytes (CD11c^+^CD83^+^, CD11c^+^CD86^+^) through flow cytometry to understand the immune response. The results demonstrated that the administration of nanovaccines (NVs) prompted immune responses towards Th1 pathway. This was indicated by notable enhancements in the production of specific antibodies, heightened cytokine levels, and a robust proliferation of lymphocytes observed in mice that received the NVs compared to control groups. Remarkably, mice vaccinated with the antigen-loaded NPs formulations exhibited considerably higher proportions of splenic dendritic cells (DCs) and T cells in comparison to those receiving the traditional adjuvant or the control groups. Incorporating HcES-24 protein into NPs effectively conferred immunity against *H. contortus*, paving the way for developing a targeted and commercial vaccine.

## 1 Introduction

*Haemonchosis*, a consequential ailment attributed to the helminth *Haemonchus contortus*, imposes substantial economic burdens and diminishes productivity in sheep and goats due to its hematophagous behavior in the abomasum (1). This nematode infiltrates its host through contaminated pasture and actively syphon blood from the abomasal region. Infected animals exhibit poor growth, dehydration, anemia, and heightened mortality rates in young lambs (2, 3). The absence of an efficacious vaccine against *H. contortus* infection has sparked keen interest among researchers in developing therapeutic interventions aimed at prevention (4). During the developmental stages of parasites like *H. contortus*, an array of antigens, referred to as excretory/secretory proteins (ESPs), are released into the host. These ESPs possess heightened immunogenic properties, rendering them promising candidates for vaccine development (5).

Helminth ESPs encompass a significant presence of CAP superfamily proteins known as activation-associated secreted proteins (ASPs) or sperm coating proteins (SCP). These proteins are pivotal in orchestrating the transition of larvae from a free living state to the parasitic stage (6). Research suggests that SCP like extracellular protein domains are also found in pathogenesis related proteins within plants, indicating potential roles in anti-fungal activities or involvement in altering cell walls post-pathogen invasion. These molecules are postulated to be involved in the onset, establishment, and perpetuation of the host–parasite relationship (7, 8). Due to their prevalence in the ESPs of third stage infective larvae (L3), SCPs are believed to play crucial roles during the shift from free living to parasitic stages upon invasion into mammalian hosts (1, 9). Notably, Na-ASP-2, an ASP, has been under exploration as a potential vaccine candidate against human *Necatoriasis* (10). Furthermore, studies on recombinant *Onchocerca volvulus* ASP-1 have demonstrated partial protective immunity in mice challenged with *O. volvulus* L3s (11).

During vaccine formulation, peptides and proteins frequently demonstrate diminished immunogenicity when administered alone (12). Previous investigations have validated that the inclusion of adjuvants substantially augment the efficacy of purified antigens, showing enhancements of up to 82% in vaccines (13). In this scenario, poly(d, l-lactide-co-glycolide) (PLGA) is extensively investigated for its adjuvant potential, representing a frequently studied polymer (14). It has received approval for sustained drug delivery in human therapeutics as it is a biodegradable and also a biocompatible polymer (15). Similarly, chitosan (CS) is a deacetylated form of chitin which is a polysaccharide present abundantly in the shells of crustaceans (16, 17). The cationic nature of PLGA and CS has been conveniently exploited for the development of particulate drug delivery systems (18, 19).

In this study, we systematically examined the immune responses elicited by HcES-24-loaded PLGA and CS NPs in a murine model. Our results demonstrated the activation of dendritic cell and T cell phenotypes, significant proliferation of splenic lymphocytes, enhanced antibody production, and increased cytokine secretion. These findings establish a pivotal framework for advancing the development of next-generation vaccines against *H. contortus*.

## 2 Materials and methods

### 2.1 Ethical approval

The handling of all animals strictly followed the guidelines stipulated by the Animal Ethics Committee at Nanjing Agricultural University, PR China. All animal experiments were conducted in compliance with the regulations established by the Animal Welfare Council of PR China. Furthermore, the experimental protocols received approval from the Science and Technology Agency of Jiangsu Province (Approval ID: SYXK (SU) 2010–0005).

### 2.2 Animals and proteins

A total of 80 female Institute of Cancer Research (ICR) mice, aged 8–10 weeks and weighing between 18 and 20 g, were acquired from the Experimental Animal Centre of Jiangsu. These mice were certified with the qualification certificate SCXK 2017–0001. The mice were kept in a controlled environment that was free from specific pathogens (SPF) and were given unrestricted access to sterilized food and water. The Molecular Parasitology and Immunology laboratory at Nanjing Agricultural University provided the purified recombinant proteins of HcES-24 and pET-32a, which were expressed in BL21 (*E. coli*) (20).

### 2.3 Preparation of rHcES-24-loaded PLGA and CS NPs

The PLGA NPs (with a molecular weight range of 40,000 to 75,000 MW, obtained from Sigma-Aldrich, St. Louis, MO, USA) were produced using the two fold emulsion method (water–oil–water), as described in a previous study (21) with specific modifications made under sterile conditions. Briefly, the inner aqueous phase consisted of the recombinant protein rHcES-24 (3.5 mg/mL) dissolved in a 6% Polyvinyl alcohol (PVA) solution from Sigma-Aldrich, St. Louis, MO, USA. The organic phase was created by dissolving 5% PLGA in methylene chloride. By utilizing ultrasonic processing, the two phases were combined to produce the primary emulsion, which consisted of water-in-oil. The emulsion was added to an external aqueous phase containing 6% PVA dissolved in deionized water to create the final emulsion (water–oil–water). The organic solvent was evaporated under magnetic stirring at a speed of 800 revolutions per minute for a duration of 4–5 h at the ambient temperature. Subsequent isolation of the resultant antigen loaded NPs involved centrifugation at 40,000 rpm for 40 min at 4°C, followed by storage in a − 80°C refrigerator until usage.

For the CS NPs (50,000–190,000 Da, Sigma-Aldrich, St. Louis, MO, USA), the ionic gelation method was applied, following previous protocols (22). Initially, 200 mg CS dissolved in a 1% acetic acid solution underwent pH adjustment to 5.0 using NaOH (2.0 N). Stepwise addition of the rHcES-24 (3.5 mg/mL) into the CS solution occurred under continuous stirring. Subsequent introduction of sodium tripolyphosphate (TPP, MW = 367.86, Aladdin, Shanghai, PR China) triggered a reaction, followed by ultra-sonication and subsequent centrifugation at 40,000 rpm for 40 min at 4°C. Next, the resultant NPs were freeze dried for 24 h preceded storage at −80°C. Blank PLGA and CS NPs were synthesized following the methodology outlined for antigen loaded counterparts, omitting the addition of the antigen (rHcES-24).

### 2.4 Preparation of antigen-loaded Freund’s adjuvant

The purified rHcES-24 (3.5 mg/mL) was solubilized in PBS and then blended with complete Freund’s adjuvant (CFA) in a 1:1 proportion. A matching volume of PBS was utilized to mix with the protein volume, establishing a control.

### 2.5 The characterization of antigen loaded PLGA and CS NPs

The protein integrity was evaluated by performing sodium dodecyl sulfate polyacrylamide gel electrophoresis (SDS-PAGE) on the loaded NPs. Additionally, the surface characteristics of the PLGA and CS NPs were observed using a cold field emission scanning electron microscope (SEM, JEOL IT-100, Japan) to determine their morphology. The PLGA and CS NPs, in a dry powder form, were affixed onto aluminum stubs, coated with platinum, and analyzed using a microscope. The supernatants collected after washing the nanoparticles (PLGA, CS) were used to measure the protein loading capacity (LC) and encapsulation efficiency (EE) using the Micro BCA™ protein assay kit (CW Biotech, Beijing, PR China), according to certain computational equations (23, 24).


EE=(total protein–unbound protein)/total protein×100%

LC=loaded protein/total mass ofNV×100%

*In vitro* assessment of recombinant HcES-24 protein release from PLGA and CS NPs involved monitoring changes in the unbound recombinant HcES-24 in the solution. Lyophilized nanoparticles (3 mg) were dispersed in 150 μL of sterile PBS (0.1 M, pH 7.4) and subjected to agitation in a shaker bath (37°C, 120 rpm). At specific time points (0, 1, 3, 5, 7, 9, 11, 13, 15, 17, 19 and 21 days), the suspension underwent centrifugation at 12,000 rpm for 15 min. Following centrifugation, 100 μL of supernatant was withdrawn and promptly replaced with an equivalent volume of fresh PBS. The concentration of free rHcES-24 protein in the supernatants was determined using a Micro BCA™ Protein Assay Kit. All experimental procedures were conducted in triplicate.

### 2.6 Mice immunization

Mice, randomly grouped into ten sets with 8 animals in each, were immunized on day 0 and ethically euthanized on the 14th day of the experiment. Control groups encompassed PBS, pET-32a vector protein, PBS + CFA, PBS + rHcES-24, PBS + CS NPs, and PBS + PLGA NPs, while experimental groups comprised of rHcES-24 + CFA, rHcES-24 + CS + PLGA NPs, rHcES-24 + CS NPs, and rHcES-24 + PLGA NPs. Using a subcutaneous (SC) injection method, mice were administered 1 mL (20 μg) of rHcES-24 protein at multiple sites, following established protocols (25). The rHcES-24 + CS + PLGA NPs group received an equivalent quantity of 10 μg of both rHcES-24 + CS NPs and rHcES-24 + PLGA NPs. A summary of the distinct animal groups and their vaccination schemes is presented in Table 1.

**Table 1:** Composition of the different materials (as vaccine) injected into ICR mice.

Groups	Inoculations	Injection at 0 Day	Aim of injection
1	PBS	1	Blank control
2	pET-32a Protein	1	Negative control
3	PBS + CFA	1	Immunogenicity of complete Freund’s adjuvant
4	PBS + rHcES-24	1	Immunogenicity of rHcES-24
5	PBS + CS	1	Immunogenicity of CS NPs
6	PBS + PLGA	1	Immunogenicity of PLGA NPs
7	rHcES-24 + CFA	1	Immunogenicity of rHcES-24 with complete Freund’s adjuvant
8	rHcES-24 + CS + PLGA	1	Comparison of immunogenicity of rHcES-24 with CS & PLGA NPs
9	rHcES-24 + CS	1	Comparison of immunogenicity of rHcES-24 with CS NPs
10	rHcES-24 + PLGA	1	Comparison of immunogenicity of rHcES-24 with PLGA NPs

### 2.7 ELISA for antibody determination in sera

Before euthanizing the mice, serum samples were collected to assess the quantities of antibodies generated, specifically IgG1, IgG2a, and IgM. The mouse ELISA kits manufactured by HengYuan in Shanghai, PR China, were used in accordance with the instructions provided by the manufacturer. In summary, 96 well microplates were covered with purified antibodies that target mouse IgG1, IgG2a, or IgM. The mouse serum samples were diluted in PBS and thereafter added to the wells. The samples were then incubated at a temperature of 37°C for a duration of 30 min. After washing with PBST, the incubation with HRP conjugated anti-mouse antibodies was performed to determine the antibody level and analyze the isotype. The experiment involved adding a 200 μL solution containing substrates A and B. The reaction was then stopped using a 2 M H_2_SO_4_ solution. Each plate comprised both positive and negative controls. The results were quantified using spectrophotometry, specifically by measuring the absorbance at a wavelength of 450 nm.

### 2.8 Th1/Th2 index calculation

Upon quantification of IgG1 and IgG2a antibodies, the Th1/Th2 indices were calculated for the four immunized groups (rHcES-24 + CFA, rHcES-24 + CS + PLGA NPs, rHcES-24 + CS NPs, rHcES-24 + PLGA NPs) using the IgG2a/IgG1 formula. A Th1/Th2 index exceeding 1 denotes a shift toward the Th1 type response associated with cellular immunity, while an index below 1 signifies a tendency toward the Th2 type response linked to humoral immunity (26).

### 2.9 Cytokine measurements

The concentrations of cytokines, namely IL-4, IL-10, IL-17, IFN-*γ*, and TGF-*β* in the serum samples obtained from various groups were determined using commercially available ELISA kits sourced from HengYuan, Shanghai, PR China, following the manufacturer’s instructions.

### 2.10 Analysis of lymphocyte proliferation

The lymphocyte proliferation assay was employed to evaluate the proliferative response of splenic lymphocytes isolated from each mouse group (27). Animals from different cohorts were euthanized in a human manner, and their spleens were extracted from the dissected animals. Subsequently, splenic lymphocytes were extracted using the Mouse Spleen Lymphocyte Isolation Kit (TBD, Tianjin, PR China) in a sterile environment. The cells were gathered and concentrated to a density of 1 × 10^7^ cells/mL in RPMI-1640 culture medium (CM). They were then left to incubate overnight in 6 well cell culture plates. Afterwards, the liquid portion that contains T cells and B cells was gathered, and the number of cells in the population was adjusted to 1 × 10^6^ cells per milliliter. The cells were subsequently placed in 96 well culture plates at a density of 1 × 10^6^ cells per well. The culture medium used was supplemented with 10% heat-inactivated fetal calf serum, 100 U/mL penicillin, and 100 mg/mL streptomycin (Gibco, Carlsbad, CA, USA). Stimulation was induced by the addition of the target protein (3 μg/mL of rHcES-24) and incubated for 72 h. Control groups, including the blank control (without rHcES-24 stimulation) and the positive control with ConA, were included on the same plate. After the incubation period, we evaluated the cell growth stimulated by rHcES-24 using the Enhanced Cell Counting Kit-8 (Beyotime) as per the instructions provided by the manufacturer. The stimulation index (SI) is determined by calculating the ratio of the mean A450 value of the test group (At) to that of the blank control group (Ac) (28).

### 2.11 Flow cytometry

The proportions of CD4^+^ and CD8^+^ T lymphocytes within the spleens of mice from different experimental groups were assessed by flow cytometry, following a previously published technique (29). At first, T cells were separated according to the procedure described in section 2.10 and then treated with anti-CD3e-APC and anti-CD4-FITC antibodies to measure the number of CD4^+^ T cells. In addition, cells were labelled with anti-CD8a-FITC antibodies to quantify the number of CD8^+^ T cells.

In order to assess the development of DCs, splenic cells were obtained from the experimental mice using the Spleen Lymphocytes Isolation Kit. The cells were grown in RPMI-1640 medium and incubated overnight. Following the incubation period, the liquid portion of the mixture was removed and the cells that were attached to the surface were carefully collected and rinsed with PBS. These cells were then stained with anti-CD11c-APC, anti-CD83-PE, and anti-CD86-PE antibodies in order to determine the percentages of cells expressing CD83^+^ and CD86^+^. Following centrifugation and washing, flow cytometry analysis was performed using FACS Caliber. The antibodies utilized in the studies were acquired from Biolegend, located in San Diego, CA, USA.

### 2.12 Statistical analysis

The experiments were replicated three times, and the findings are presented as mean values with standard error of the mean. Group differences were determined using two-way ANOVA followed by Tukey’s *post-hoc* test (*p < 0.05, **p < 0.01, and ***p < 0.001). Data analysis for FACS was performed using Flow Jo version 10 software and employed GrpahPad Prism 8 to assess other experimental data.

## 3 Results

### 3.1 Physical properties of antigen-loaded NPs

**Figure 1:**
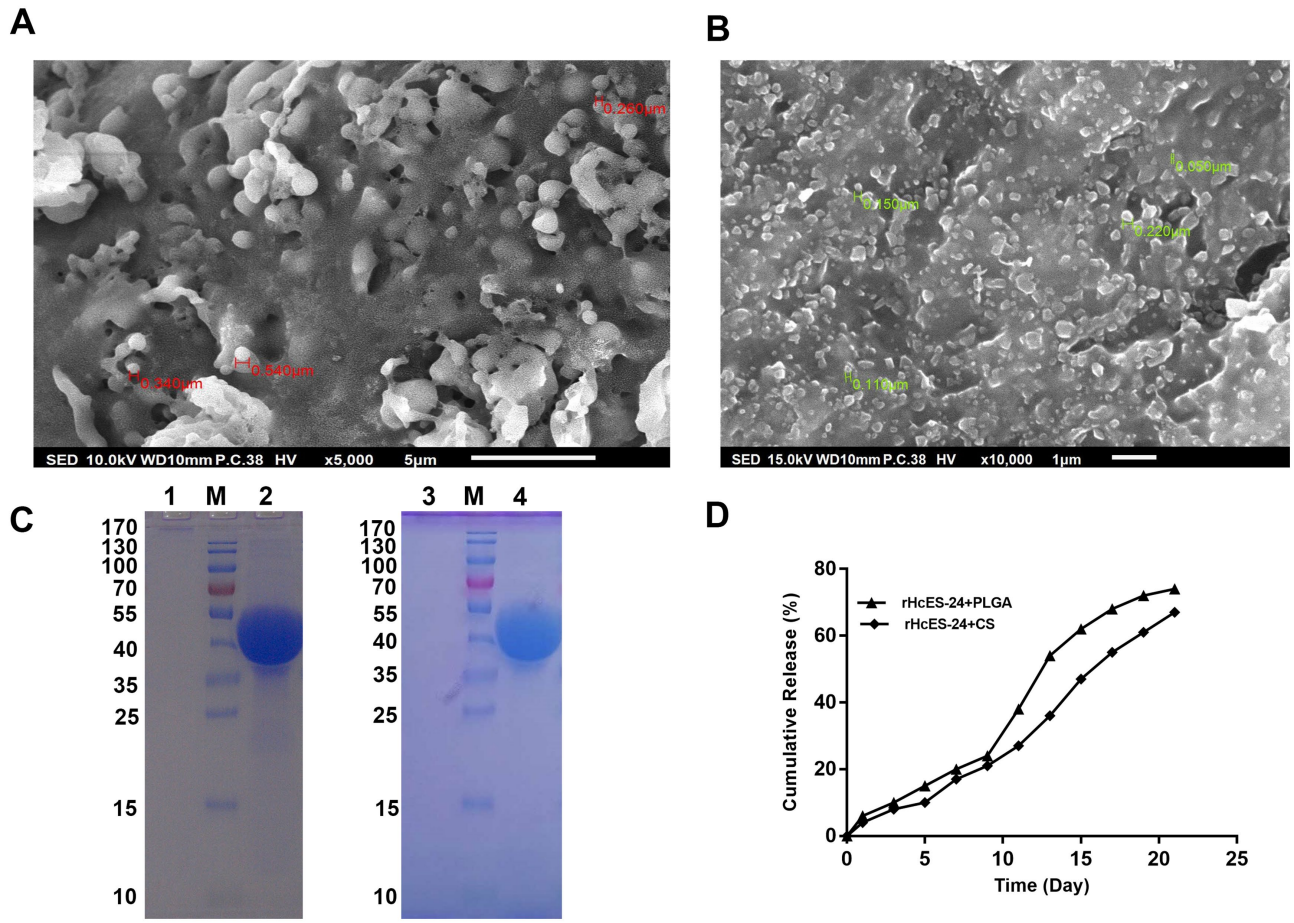
The experiment utilized SEM to analyze the surface structure of different antigen-loaded systems, seeing nanoparticles (NPs) at a magnification of 10,000x. (A) Displayed the SEM image of rHcES-24 + PLGA NPs, whereas (B) showed the SEM results of rHcES-24 + CS NPs. In addition, the study employed SDS-PAGE analysis using a 12% separating gel to examine the integrity of rHcES-24 with PLGA and CS NPs. The gel lanes were labelled as follows: Lane M represented the usual protein molecular weight marker, Lane 1 showed PLGA NPs without rHcES-24, Lane 2 displayed PLGA NPs with bound rHcES-24, Lane 3 depicted CS NPs without rHcES-24, and Lane 4 exhibited CS NPs with bound rHcES-24 (C). In (D), the cumulative release of HcES-24 from NPs.

SEM analysis showed that both PLGA and CS NPs had a smooth surface. The size of rHcES-24 + PLGA NPs varied between 502 nm and 3.202 μm, as shown in Figure 1A. On the other hand, the size of rHcES-24 + CS NPs ranged from 50 nm to 220 nm, as depicted in Figure 1B.

The SDS-PAGE examination was performed using a 12% separating gel to assess the integrity of rHcES-24 and PLGA or CS NPs. The results showed clear bands at around 42 kDa for both rHcES-24 + PLGA NPs and rHcES-24 + CS NPs. In contrast, PBS + PLGA NPs and PBS + CS NPs did not show any bands in the same range (Figure 1C).

*In vitro* evaluation of protein release kinetics was conducted through a cumulative release assay, as illustrated in Figure 1D. Over a 21-day duration, PLGA NPs released approximately 78% of the antigen, while CS NPs exhibited a release of around 63%.

The unbound rHcES-24 protein trapped within the NPs was quantified using the Micro-BCA™ protein assay kit. The outcomes indicated an encapsulation efficiency (EE) of 77.6% for rHcES-24 within PLGA NPs and 72.8% within CS NPs. Moreover, PLGA NPs contained approximately 38.8% of loaded protein, while CS NPs encapsulated around 36.4% (Table 2).

**Table 2:** Properties of antigen (rHcES-24) loaded PLGA and CS NPs.

NPs	Size (μm)	LCa	EEb
rHcES-24 + PLGA	100 ± 40	38.8	77.6
rHcES-24 + CS	115 + 35	36.4	72.8

LCa = (total protein − unbound protein)/total dry weight of Nano-vaccine × 100%. EEb = (total protein − unbound protein)/total protein × 100%.

### 3.2 NPs increased the secretion levels of serum antibodies

**Figure 2:**
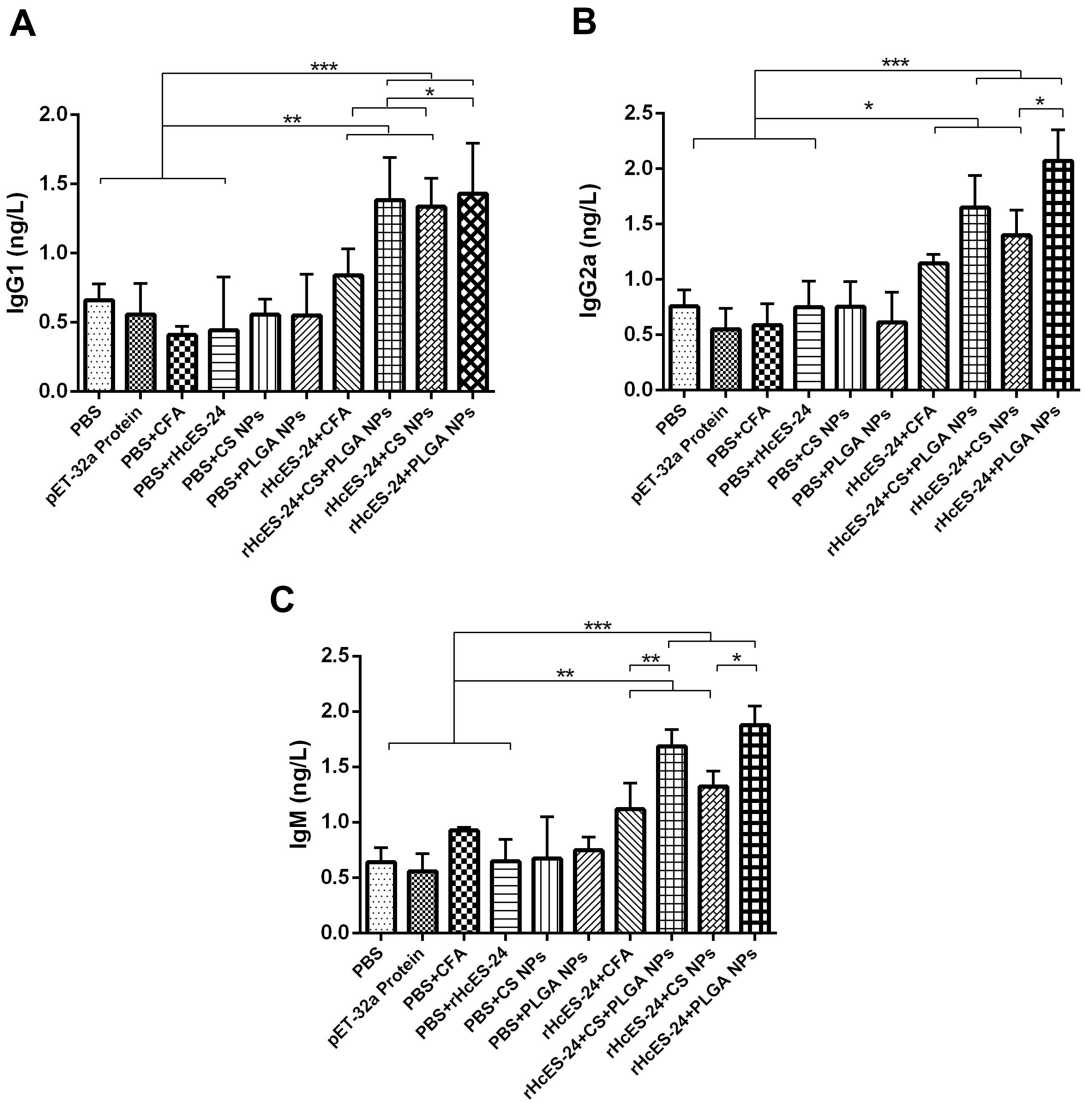
The effect of antigen-loaded nanoparticles (NPs) on the immunological response in ICR mice was assessed using ELISA. The mice were immunized by receiving a single injection under the skin on day 0, either with or without antigen-loaded nanoparticles. On the 14th day after immunization, blood samples were obtained to examine the levels of antibodies in the serum, specifically targeting antigen-specific IgG1 (A), IgG2a (B), and IgM (C) antibodies. The data reported in this study are the outcomes of three separate studies. Statistical significance is indicated by the symbols (*p < 0.05, **p < 0.01, ***p < 0.001).

The ELISA performed on serum samples obtained from different groups of mice showed clear differences in the levels of IgG1, IgG2a, and IgM, as shown in Figure 2. Mice that received vaccinations with rHcES-24 + CFA, rHcES-24 + CS + PLGA NPs, rHcES-24 + CS NPs, and rHcES-24 + PLGA NPs showed significantly elevated levels of IgG1 compared to the PBS group (control) and significantly higher levels than the pET-32a protein, PBS + CFA, and PBS + rHcES-24 groups (**p < 0.01, ***p < 0.001). The group treated with rHcES-24 + PLGA NPs showed significantly increased levels of IgG1 compared to the groups treated with rHcES-24 + CFA and rHcES-24 + CS NPs (*p < 0.05) (Figure 2A).

Additionally, the mice that were immunized with formulations of rHcES-24 + CFA, rHcES-24 + CS + PLGA NPs, rHcES-24 + CS NPs, and rHcES-24 + PLGA NPs showed a significant increase in the secretion of IgG2a and IgM compared to the groups that received PBS, pET-32a protein, PBS + CFA, and PBS + rHcES-24 (*p < 0.05, **p < 0.01, ***p < 0.001). The group treated with rHcES-24 + PLGA NPs showed significantly greater levels of IgG2a and IgM compared to the groups treated with rHcES-24 + CFA, rHcES-24 + CS + PLGA NPs, and rHcES-24 + CS NPs (*p < 0.05, **p < 0.01) (Figures 2B,C).

### 3.3 NPs modulated cytokines production

**Figure 3:**
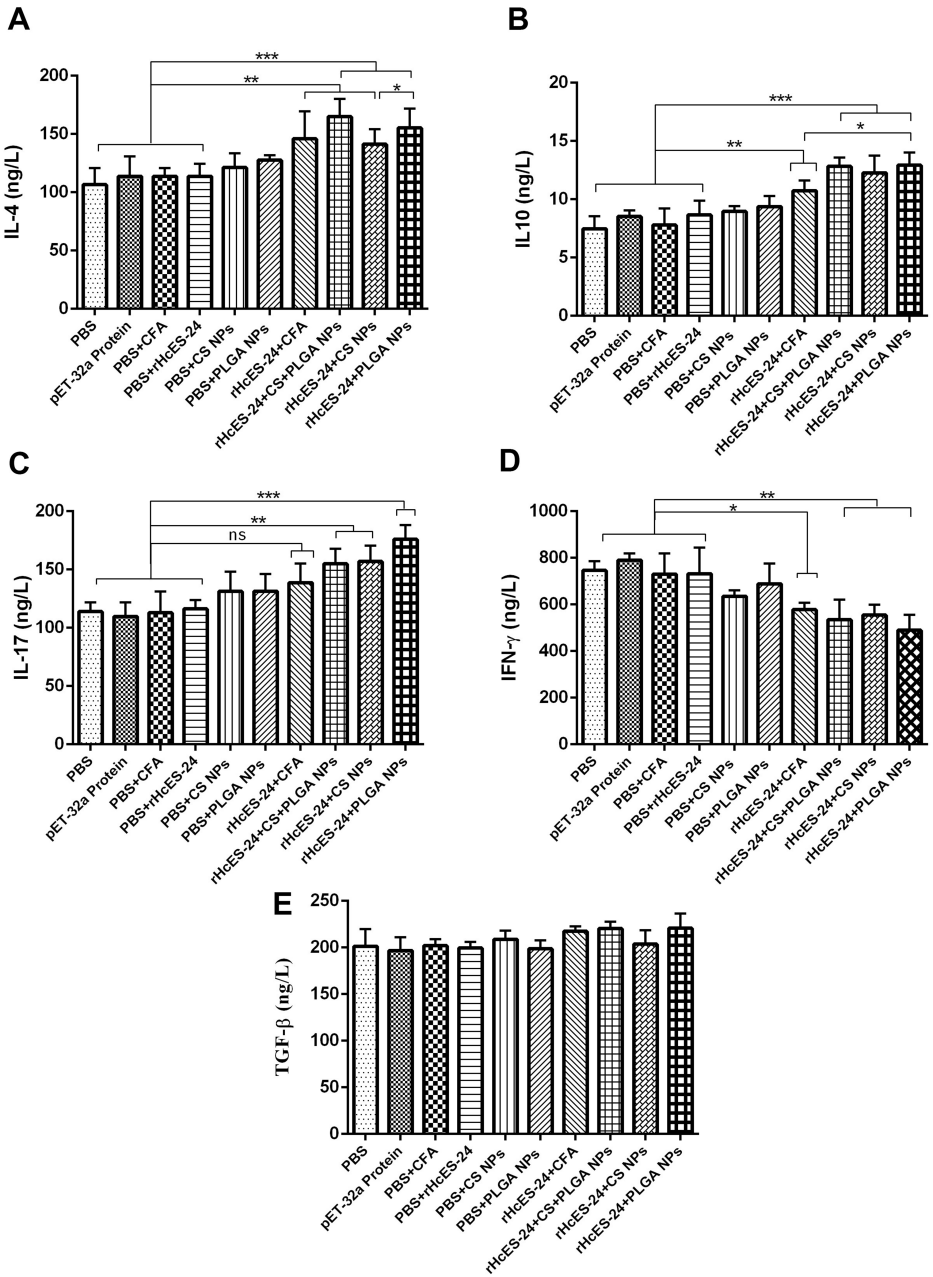
The effects of different methods of delivering antigens on the measurement of numerous cytokines were assessed using ELISA in several experimental animal groups. More precisely, a group of ICR mice (*n* = 8) received a single immunization process as described in the experimental section. Before the mice were sacrificed, serum samples were collected and analyzed on the 14th day using ELISA to measure the levels of different cytokines, namely: (A) IL-4, (B) IL-10, (C) IL-17, (D) IFN-γ, (E) TGF-*β*. The data provided are from separate studies, each performed three times, and the significance levels are indicated as (*p < 0.05, **p < 0.01, ***p < 0.001).

The observed outcomes demonstrated significantly increased levels of IL-4 in groups treated with rHcES-24 + CFA, rHcES-24 + CS + PLGA NPs, rHcES-24 + CS NPs, and rHcES-24 + PLGA NPs compared to the PBS, pET-32a protein, PBS + CFA, and PBS + rHcES-24 groups (**p < 0.01, ***p < 0.001). Additionally, the rHcES-24 + PLGA NPs and rHcES-24 + CS + PLGA NPs groups displayed higher IL-4 levels than the rHcES-24 + CS NPs and rHcES-24 + CFA groups (*p < 0.05) (Figure 3A).

Moreover, sera from mice immunized with rHcES-24 + CFA, rHcES-24 + CS + PLGA NPs, rHcES-24 + CS NPs, and rHcES-24 + PLGA NPs displayed significantly elevated concentrations of IL-10 compared to the PBS, pET-32a protein, PBS + CFA, and PBS + rHcES-24 groups (**p < 0.01, ***p < 0.001). The rHcES-24 + PLGA NPs, rHcES-24 + CS NPs, and rHcES-24 + CS + PLGA NPs groups also exhibited notably increased IL-10 levels compared to the rHcES-24 + CFA group (*p < 0.05). However, no significant differences were observed in IL-10 secretions among the rHcES-24 + PLGA NPs, rHcES-24 + CS NPs, and rHcES-24 + CS + PLGA NPs groups (Figure 3B).

Regarding IL-17, elevated levels were noted in the rHcES-24 + CS + PLGA NPs, rHcES-24 + CS NPs, and rHcES-24 + PLGA NPs groups compared to the PBS group (**p < 0.01, ***p < 0.001). Non-significant differences were observed between the rHcES-24 + CFA group and the control groups, although, it was secreted in a bit higher quantity compared to control groups. Additionally, rHcES-24 + PLGA NPs induced higher IL-17 levels compared to rHcES-24 + CS + PLGA NPs and rHcES-24 + CS NPs (*p < 0.05, Figure 3C).

There was a distinct reduction in IFN-*γ* secretion in the rHcES-24 + CFA, rHcES-24 + CS + PLGA NPs, rHcES-24 + CS NPs, and rHcES-24 + PLGA NPs groups compared to the PBS group (*p < 0.05, **p < 0.01). Furthermore, compared to rHcES-24 + CFA, the levels of IFN-γ secreted by rHcES-24 + CS + PLGA NPs, rHcES-24 + CS NPs, and rHcES-24 + PLGA NPs groups were considerably low (*p < 0.05). However, no significant difference was observed between the rHcES-24 + CFA and rHcES-24 + CS NPs groups (Figure 3D).

The levels of TGF-*β* secretion showed no significant variations across the groups (Figure 3E).

### 3.4 NPs augmented the proliferation of splenic lymphocyte

**Figure 4:**
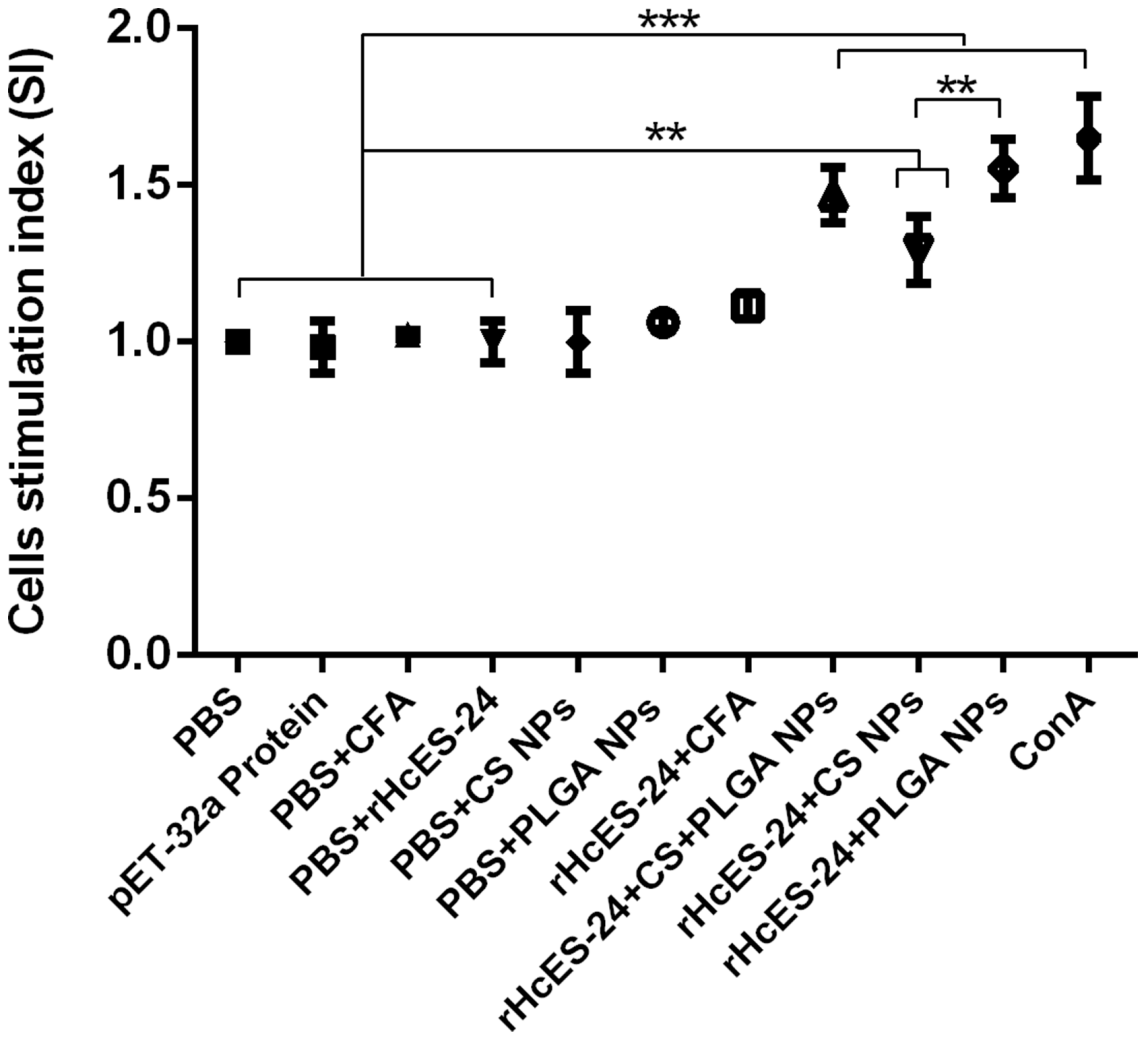
The proliferation indices of splenic lymphocytes were assessed *in vitro* using spectrophotometric analysis at an optical density of 450. Splenocytes obtained from different immunized animals were stimulated with varied treatments. The data presented are from three separate trials and show the mean values ± SEM (**p < 0.01, ***p < 0.001).

The splenic lymphocytes isolated from mice across diverse experimental groups on the 14th day were analyzed for their specific proliferation response to rHcES-24, presented as Stimulation Index (SI) values in Figure 4. Particularly, both the ConA (positive control) and the rHcES-24 + PLGA NPs groups exhibited significantly higher proliferation in comparison to the PBS, pET-32a protein, PBS + CFA, and PBS + rHcES-24 groups (***p < 0.001). Moreover, the rHcES-24 + CFA, rHcES-24 + CS + PLGA NPs, and rHcES-24 + CS NPs groups demonstrated elevated proliferation levels compared to the PBS, pET-32a protein, PBS + CFA, and PBS + rHcES-24 groups (**p < 0.01, ***p < 0.001). Importantly, when contrasted with rHcES-24 + CFA; the ConA, rHcES-24 + PLGA NPs, and rHcES-24 + CS + PLGA NPs groups displayed substantially heightened SI values (***p < 0.001). Additionally, the rHcES-24 + CS NPs group also showcased increased SI values relative to rHcES-24 + CFA (*p < 0.05).

### 3.5 Polarization of T cells

The tabular representation depicting the ratio of IgG2a/IgG1 reveals a prominent Th1 skewed immune response across all vaccinated groups (Table 3).

**Table 3:** IgG2a/IgG1 ratios in four different groups that received the same volumes of antigenic material and NPs.

Groups	rHcES-24 + CFA	rHcES-24 + CS + PLGA	rHcES-24 + CS	rHcES-24 + PLGA
Mean of IgG2a	172.7216	188.3163	175.2943	194.406
Mean of IgG1	46.477	48.59	44.924	50.06
Ratio of IgG2a/IgG1	3.716	3.875	3.902	3.883

The secretion levels of IgG1 and IgG2a were detected by ELISA.

### 3.6 NPs activate the propagation of T cells

**Figure 5:**
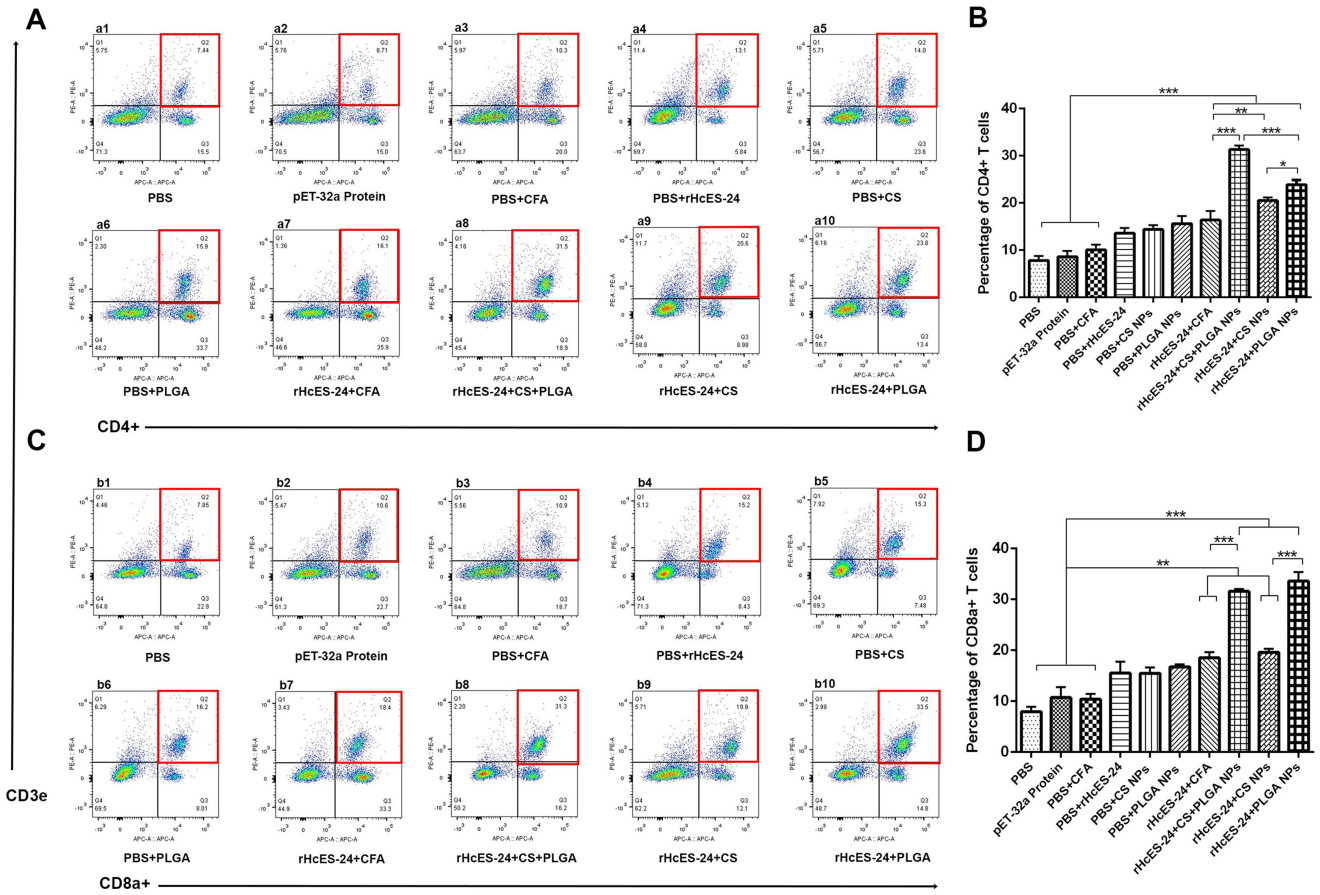
Flow cytometry was used to determine the percentages of CD4^+^ and CD8^+^ T cells in all mice groups in subsection of (A,B). The bar graphs shown in (C,D) represent the proportions of these T cell subgroups in 10 different groups. (A,B) Demonstrate alterations in the ratios of CD4^+^ and CD8^+^ T cells following various therapies. The groups were categorized as follows: a1, b1: Control group treated with PBS (phosphate-buffered saline); a2, b2: Group treated with pET-32a vector protein; a3, b3: Group treated with PBS and CFA (complete Freund’s adjuvant); a4, b4: Group treated with PBS and rHcES-24; a5, b5: Group treated with PBS and CS NPs (chitosan nanoparticles); a6, b6: Group treated with PBS and PLGA NPs (poly(lactic-co-glycolic acid) nanoparticles); a7, b7: Group treated with rHcES-24 and CFA; a8, b8: Group treated with rHcES-24, CS, and PLGA NPs; a9, b9: Group treated with rHcES-24 and CS NPs; a10, b10: Group treated with rHcES-24 and PLGA NPs. The results presented here are from one independent experiment that is consistent with three other experiments. These results show statistical significance at different levels: (*p < 0.05, **p < 0.01, ***p < 0.001).

The flow cytometry analysis of lymphocytes from distinct experimental groups revealed percentages of CD4^+^ and CD8a^+^ T cells, presented in Figure 5. Marked elevation in CD3e^+^CD4^+^ cell levels were observed in the rHcES-24 + CS + PLGA NPs, rHcES-24 + CS NPs, and rHcES-24 + PLGA NPs groups compared to the PBS group (***p < 0.001). Relative to control groups (pET-32a protein, PBS + CFA, and PBS + rHcES-24), the rHcES-24 + CFA, rHcES-24 + CS + PLGA NPs, rHcES-24 + CS NPs, and rHcES-24 + PLGA NPs groups displayed significantly higher CD3e^+^CD4^+^ cell percentages (**p < 0.01, ***p < 0.001). Specifically, the rHcES-24 + CS + PLGA NPs group exhibited a substantial rise in CD3e^+^CD4^+^ cells compared to the rHcES-24 + CFA, rHcES-24 + CS NPs, and rHcES-24 + PLGA NPs groups (***p < 0.01). Furthermore, the CD4^+^ T cell proliferation in the rHcES-24 + PLGA NPs group surpassed that of the rHcES-24 + CFA and rHcES-24 + CS NPs groups (*p < 0.05, Figures 5A,B).

In comparison to the PBS group, significantly higher percentages of CD3e^+^CD8a^+^ cells were observed in the rHcES-24 + CFA, rHcES-24 + CS + PLGA NPs, rHcES-24 + CS NPs, and rHcES-24 + PLGA NPs groups, as well as when compared to the pET-32a protein, PBS + CFA, and PBS + rHcES-24 groups (**p < 0.01, ***p < 0.001). Notably, the rHcES-24 + CS + PLGA NPs and rHcES-24 + PLGA NPs groups exhibited significantly higher CD3e^+^CD8a^+^ cell percentages compared to the rHcES-24 + CFA and rHcES-24 + CS NPs groups (***p < 0.001). Moreover, the rHcES-24 + PLGA NPs group displayed the highest percentages of these cells (Figures 5C,D).

### 3.7 NPs augmented the number of splenic DCs

**Figure 6:**
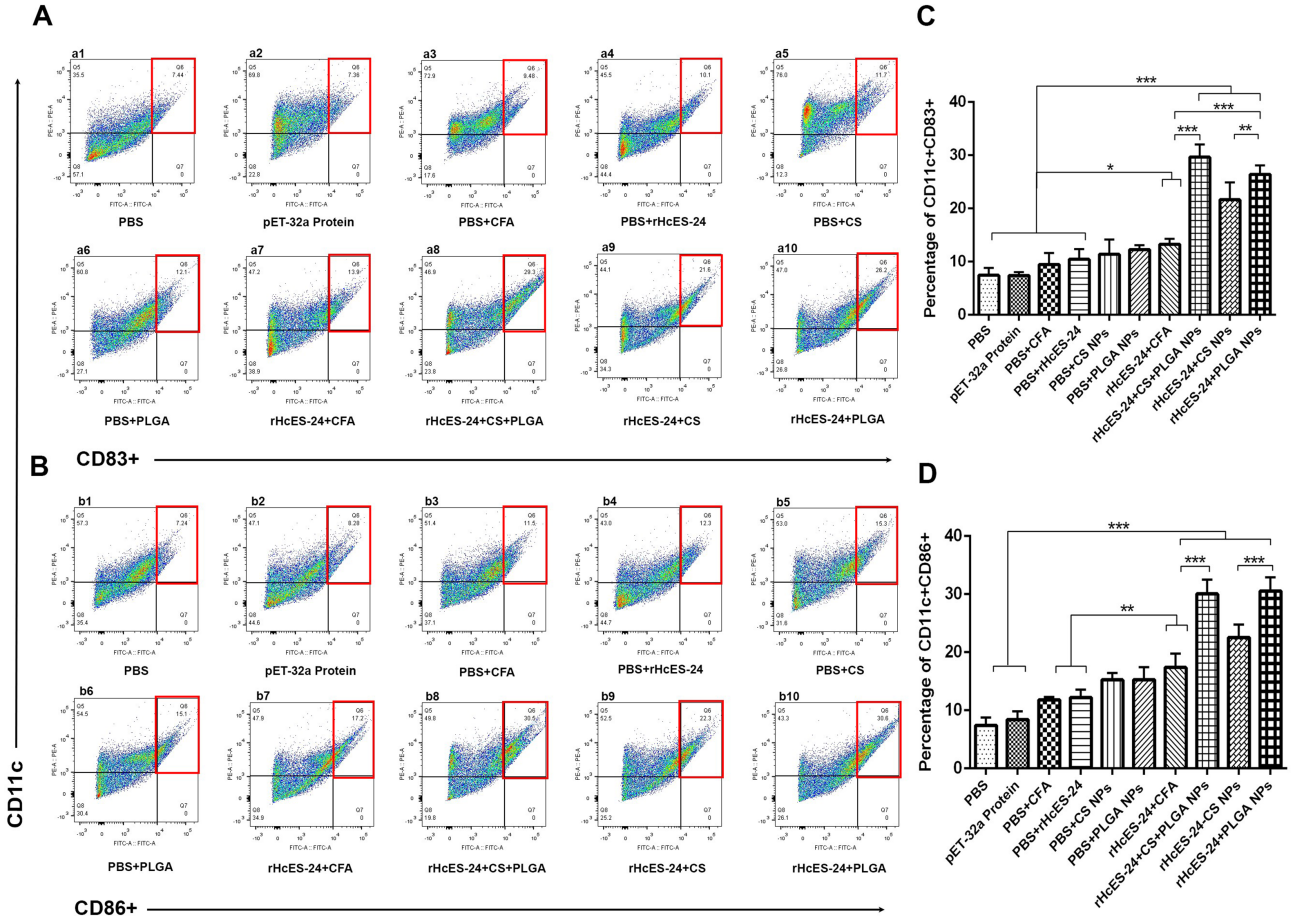
The study sought to evaluate the impact of various therapies on the development of splenic dendritic cells (DCs) and their ability to deliver antigens. Flow cytometry was employed to analyze the expression levels of CD11c^+^CD83^+^ (A) and CD11c^+^CD86^+^ (B) in splenic DCs across 10 experimental groups. The bar graphs (C,D) display the average values with standard error of the mean (SEM) and represent the outcomes of three separate trials. The significance levels are indicated as (*p < 0.05, **p < 0.01, ***p < 0.001).

The flow cytometry was employed to examine the interaction and uptake of antigenic NPs by DCs in the spleen. The frequencies of CD83^+^ and CD86^+^ lymphocytes were evaluated in 10 different groups of mice (Figure 6). Results showed significant increases in the proportion of CD11c^+^CD83^+^ cells in the rHcES-24 + CFA, rHcES-24 + CS + PLGA NPs, rHcES-24 + CS NPs, and rHcES-24 + PLGA NPs groups compared to the PBS, pET-32a protein, PBS + CFA, and PBS + rHcES-24 groups (*p < 0.05, ***p < 0.001). The groups treated with rHcES-24 + CS + PLGA NPs, rHcES-24 + CS NPs, and rHcES-24 + PLGA NPs showed larger percentages of CD11c^+^CD83^+^ cells compared to the rHcES-24 + CFA group (***p < 0.001). The group treated with rHcES-24 + CS + PLGA NPs exhibited significantly higher levels of CD11c^+^CD83^+^ cells compared to the groups treated with rHcES-24 + CFA, rHcES-24 + CS NPs, and rHcES-24 + PLGA NPs (*p < 0.05, **p < 0.01, ***p < 0.001). In addition, mice treated with rHcES-24 + PLGA NPs showed a higher abundance of splenic DCs compared to the rHcES-24 + CS NPs group (statistically significant at **p < 0.01, as shown in Figure 6A).

Significant increases in the percentages of CD11c^+^CD86^+^ cells were detected in the groups treated with rHcES-24 + CFA, rHcES-24 + CS + PLGA NPs, rHcES-24 + CS NPs, and rHcES-24 + PLGA NPs, compared to the groups treated with PBS, pET-32a protein, PBS + CFA, and PBS + rHcES-24 (**p < 0.01, ***p < 0.001). In addition, the groups treated with rHcES-24 + CS + PLGA NPs, rHcES-24 + CS NPs, and rHcES-24 + PLGA NPs showed significantly greater percentages of CD11c^+^CD86^+^ cells compared to the rHcES-24 + CFA group (**p < 0.01, ***p < 0.001). The groups treated with rHcES-24 + CS + PLGA NPs and rHcES-24 + PLGA NPs showed significantly higher percentages of CD11c^+^CD86^+^ cells compared to the group treated with rHcES-24 + CS NPs (***p < 0.001, Figure 6B).

## 4 Discussion

Sperm coating proteins (SCPs) are an integral aspect of HcESPs, abundantly expressed during *H. contortus*’ blood feeding phases (9, 30, 31). They are widespread among diverse organisms, including arthropods, flukes, nematodes, and plants. SCPs possess immunogenic properties, prompting their exploration as a potential vaccine candidate against *Necator americanus*-induced diseases in humans (8, 32). Previous research has effectively identified SCP proteins in the L4, L5, and late adult stages of *H. contortus*, highlighting their probable role in the pathogenesis of this hematophagous parasite (9). This study aims to examine the immunological attributes of rHcES-24, encapsulated within PLGA and CS NPs, in a murine model as a potential strategy against *H. contortus* infection.

Two polymers extensively investigated for vaccine delivery are PLGA and CS, renowned for their intrinsic safety, biodegradability, and applicability in formulating NPs. PLGA, a longstanding choice for controlled drug release, serves as an ideal candidate for encapsulating antigens (33, 34). These particles exhibit notable resistance to destabilization induced by salt and pH variations, gradually releasing their payload contingent upon the polymer’s hydrolysis rate (35). Conversely, CS, a water-soluble polymer, is commonly synthesized via ionic complexation or spray drying methods, resulting in positively charged particles (16). CS particles are acknowledged for their mucoadhesive properties and their ability to traverse epithelial barriers, presenting promising prospects in vaccine delivery systems.

In this study, SDS-PAGE was utilized to assess the stability of the polymers and proteins within the nanoactivating material. The findings from this complementary technique demonstrated that the structural integrity of the recombinant protein and all polymers persisted subsequent to the formulation of NPs (Figure 1C). As a result, these NPs loaded with antigen exhibited adequacy for further progression into subsequent *in vitro* and *in vivo* biological experimentation.

The importance of particulate adjuvants’ dimensions on their adjuvanticity and resulting immunogenic variation is well acknowledged. Hence, this study placed particular emphasis on the size aspect. Generally, NPs resembling pathogen sizes are efficiently recognized by antigen-presenting cells (APCs), prompting to induce an immune response (36). Preferentially, DCs internalize particles of virus like dimensions (20–200 nanometers), while larger particles (0.5–5 micrometers) are favored by macrophages (37). However, discrepancies persist among various studies regarding the optimal NPs size for vaccine efficacy (38). In this investigation, we employed an appropriate sized antigen loaded NPs to be readily identified by macrophages and other immune cells. This characteristic underscores their potential for effective interaction and uptake by immune cells, influencing their immunogenic potential.

In addition to the functions attributed to cellular immunity, specific antibodies have the capacity to hinder the parasite’s binding to host cell receptors and eliminate intracellular parasites by coating them with macrophages (39). Consequently, consistent with prior research (40, 41), mice that received injections containing rHcES-24 + CS + PLGA NPs and rHcES-24 + PLGA NPs demonstrated elevated levels of IgG1, IgG2a, and IgM compared to groups receiving rHcES-24 + CS NPs and the control.

Moreover, immunizing mice with NVs (rHcES-24 + CS NPs, rHcES-24 + PLGA NPs, rHcES-24 + CS + PLGA NPs) prompted the release of both IgG2a (indicative of Th1 and cellular response) and IgG1 antibodies (indicative of Th2 immune response). The amplified IgG2a production post-vaccination is associated with heightened uptake of particles containing the protein and adjuvant by DCs (42, 43). This highlights the potential of PLGA and CS as promising alternatives, potentially contributing significantly to inducing cellular immunity mediated by Th1 immune response. The reported finding was aligning consistently with the observed response in case of lymphocyte proliferation.

Natural killer (NK) cells and T cells are responsible for generating IFN-*γ*, a pivotal cytokine essential for host survival that triggers various intracellular mechanisms to restrain parasite replication (44). The combined action of IFN-γ and IL-10 also contributes to preventing parasitic invasion (45). IL-10 is integral in preserving host homeostasis, operating at localized and systemic levels. It maintains a delicate equilibrium between pro inflammatory and anti-inflammatory immune responses, crucial for the effective elimination of invading pathogens (46). In the context of the current study, all investigated antigen delivery systems (rHcES-24 + CFA, rHcES-24 + CS NPs, rHcES-24 + CS + PLGA NPs, rHcES-24 + PLGA NPs) induced a significant upsurge in IL-10 secretion. This heightened cytokine production proved indispensable for the sustained survival of immunized mice, underscoring the importance of these immune responses in bolstering the host’s defense mechanisms.

It is generally acknowledged that the primary protective immune response against helminths such as *H. contortus*, characterized by the secretion of IL-4 and IL-5 (47). In this study, our findings revealed that rHcES-24 entrapped in NPs could stimulate the secretion of IL-4, suggesting its potential in initiating a strong immune response against *H. contortus* infection. IL-17, a cytokine produced by Th17 cells regulating inflammation (48), was previously observed to be increased by HcESPs in our research. Our current investigation also demonstrated heightened IL-17 production in response to rHcES-24, implying its potential contribution within the realm of HcESPs. Considering the comprehensive cytokine profile findings, it is plausible that rHcES-24, when encapsulated in PLGA and CS NPs, may play a significant role in enhancing immunity and conferring protection against *H. contortus* infection.

In the current study, mice subjected to vaccination utilizing four diverse antigen delivery systems (rHcES-24 + CFA, rHcES-24 + CS NPs, rHcES-24 + CS + PLGA NPs, rHcES-24 + PLGA NPs) exhibited significant proliferation of splenocytes upon exposure to rHcES-24 that have adjuvant effect of PLGA and CS, contrasting with the control groups. This outcome strongly indicates that the immunization involving rHcES-24 effectively stimulated robust cellular immune responses against this specific helminth.

CD4^+^ T cells are essential for effective anti-tumor immunity, as evidenced in experimental studies where the absence of CD4^+^ T helper cells led to diminished functionality of CD8^+^ lymphocytes. The combined action of CD8^+^ T cells with CD4^+^ T cells is synergistic in conferring protection (49). Given the intracellular nature of *H. contortus*, a specific cellular immune response especially a robust CD4^+^CD8^+^ T cell mediated response plays a crucial regulatory role against the development and spread of *H. contortus* infection (41). In our recent investigation, flow cytometry analysis revealed a noteworthy augmentation in CD4^+^ and CD8^+^ T cells among mice immunized with rHcES-24 encapsulated NPs compared to control groups. This finding strongly indicates the activation of cellular immune responses subsequent to immunization with rHcES-24 encapsulated NPs.

NPs can undergo diverse modifications, including the incorporation or targeting of specific proteins, alteration of surface charge, and manipulation of other properties to enhance their interaction with APCs such as monocytes and DCs (50). DCs, encompassing various subtypes, play a pivotal role in coordinating both innate and adaptive immune responses, making them a crucial target for vaccine development (51). The effective delivery of antigens and adjuvants to distinct DC subsets is an active area of research aimed at optimizing both humoral and cellular vaccine responses. We analyzed the proportions of different DC phenotypes isolated from the spleens of experimental mice. Notably, mice vaccinated with rHcES-24 entrapped in NPs exhibited significantly elevated percentages of splenic DCs (***p > 0.001) compared to control groups. This notable observation suggests that the presence of this molecule (rHcES-24) potentially contributes to the proliferation and maturation of immune cells, potentially counteracting the pathogenesis associated with *H. contortus*.

## 5 Summary

In this investigation, we have demonstrated that HcES-24, when encapsulated within PLGA and CS NPs, exhibits potential as a nanovaccine candidate. The results showed its capacity to activate immune responses with notable increase in serum antibodies, cytokines, and the proliferation of T cells, dendritic cells, and splenic lymphocytes. With the immune adjuvant properties of PLGA and CS, HcES-24 holds promise for the development of an effective nanovaccine against *H. contortus* infection.

## Data Availability

The original contributions presented in the study are included in the article/supplementary material, further inquiries can be directed to the corresponding author.
